# Detection of *Campylobacter concisus* and Other *Campylobacter* Species in Colonic Biopsies from Adults with Ulcerative Colitis

**DOI:** 10.1371/journal.pone.0021490

**Published:** 2011-06-27

**Authors:** Indrani Mukhopadhya, John M. Thomson, Richard Hansen, Susan H. Berry, Emad M. El-Omar, Georgina L. Hold

**Affiliations:** Gastrointestinal Research Group, Division of Applied Medicine, University of Aberdeen, Foresterhill, Aberdeen, United Kingdom; Charité, Campus Benjamin Franklin, Germany

## Abstract

**Introduction:**

The critical role of bacteria in the pathogenesis of ulcerative colitis (UC) is well recognized, but an individual causative microorganism has not been singled out so far. *Campylobacter concisus* and other non*-jejuni* species of *Campylobacter* have been implicated as putative aetiological agents in inflammatory bowel disease in children, but such studies have not been addressed in adults. This study investigated the prevalence of *Campylobacter* species in colonic biopsy samples from adults with UC and healthy controls.

**Methods:**

Adult patients who were undergoing diagnostic colonoscopy were recruited for the study, which included 69 patients with histologically proven UC and 65 healthy controls. DNA was extracted from the biopsy samples and subjected to *Campylobacter* genus specific and *Campylobacter concisus* specific polymerase chain reaction and sequencing.

**Results:**

Detection of all *Campylobacter* DNA utilising genus specific primers was significantly higher in cases of UC, with a prevalence of 73.9% (51/69) compared to 23.1% (15/65) in controls (p = 0.0001). Nested PCR for *C. concisus* DNA was positive in 33.3% (23/69) of biopsy samples from subjects with UC, which was significantly higher than the prevalence rate of 10.8% (7/65) from controls (p = 0.0019). Sequencing of the remaining *Campylobacter* positive samples revealed that *Campylobacter ureolyticus* was positive in 21.7% (15/69) of samples from UC subjects as opposed to 3.1% (2/65) in controls (p = 0.0013). Mixed *Campylobacter* species were more common in UC patients, 20.3% (14/69) as compared to controls 4.6% (3/65) (p = 0.0084).

**Conclusion:**

The higher prevalence of *Campylobacter* genus and more specifically *C. concisus* and *C. ureolyticus* in biopsy samples from adults with UC suggests these genera of bacteria may be involved in the chronic inflammation that is characteristically seen in UC. To the best of our knowledge this is the first report of this association of *C. concisus* and *C. ureolyticus* with UC in adults.

## Introduction

Ulcerative colitis (UC) and Crohn's disease (CD) are chronic diseases of the gastrointestinal tract that together are usually referred to as inflammatory bowel disease (IBD). During the last two decades there has been a significant increase in IBD associated hospitalization, outlining the enormous economic impact to health services [1]. It has been projected that at anytime up to 240,000 people are affected by IBD in the UK [2]. More specifically, the epidemiological pattern of UC has changed significantly with an increased incidence estimated at 10–20 per 100 000 per year. The incidence rate of juvenile onset UC has also risen in Scotland over the last twenty years, which translates into a longer period of monitored healthcare for affected individuals [3], [4], [5].

Ulcerative colitis has been traditionally observed in developed societies but with increasing westernization its prevalence has been on the rise in developing countries as well [6], [7]. The current paradigm of the pathogenesis of UC revolves around an aberrant host immune response that is triggered by a poorly understood interaction between the microbiome and host genetic defects involved in the identification and clearance of microbes [8]. The genetic associations with UC are not as strong as CD suggesting a greater role of luminal factors influencing its pathogenesis. It is postulated that ‘dysbiosis’ or an imbalance between protective and harmful components of the luminal microbiota in favour of the latter plays a critical role in initiating and possibly perpetuating inflammation in UC [9].

Recent methodological advances in studying the gut microbiome have made clear distinctions between the mucosa-associated and faecal populations [10], [11]. It is suggested that in healthy individuals the mucosal microbiome forms a synergistic and stable interaction with the host immune system, while the luminal or faecal microbiome varies based on diet or other environmental factors. This distinction is critical as it is therefore more likely that mucosa-associated bacteria will have the ability and proximity to invade the protective mucous layer and the intestinal epithelial barrier. *Helicobacter* and *Bacteroides* species are two groups of mucosa-associated bacteria that have been implicated in the pathogenesis of UC [12], [13], [14]. A recent epidemiological study demonstrated an increased risk of IBD in individuals with an episode of *Campylobacter* or *Salmonella* gastroenteritis, suggesting that infection with particular bacteria may trigger the process that ultimately leads to the chronic inflammation of IBD [15]. Other population-based studies have however refuted these findings and suggested that this association is perhaps a result of detection bias [16]. More recently, the role of non-*jejuni Campylobacter* species have evoked attention, predominantly in paediatric Crohn's disease [17], [18].

The members of the *Campylobacter* genus comprise Gram negative, spiral, microaerophilic bacteria that reside in the small or large intestine of humans and animals. At least a dozen species of *Campylobacter* have been associated with human disease, with *Campylobacter jejuni* and *Campylobacter coli* the most commonly isolated strains. However, newer information suggests that non*- jejuni Campylobacter* species, most specifically *Campylobacter concisus*, may be responsible for infective gastroenteritis and septicaemia in children [19]. This species has also been isolated from stool samples of immunocompromised patients with diarrhoea suggesting that it may even be an opportunistic pathogen [20]. The recent reclassification of *Campylobacter ureolyticus* has added another species in the broader genus of *Campylobacter* having previously been within the *Bacteroides* [21]. The identification of this species in the faeces of subjects presenting with gastroenteritis suggests that it may also be an emerging enteric pathogen [22]. It appears that non*-jejuni Campylobacter* species are increasingly being identified as potential gastrointestinal pathogens. This study has for the first time aimed to delineate these mucosa-associated bacteria in biopsy samples from adults with ulcerative colitis.

## Methods

### Study Subjects

Patients were recruited from the department of gastroenterology at Aberdeen Royal Infirmary. These subjects were recruited for a previous study looking at the role of enterohepatic *Helicobacter* in UC [14]. Sixty-nine patients with a primary diagnosis of UC on the basis of a histological diagnosis from colonoscopic biopsies were recruited and assessed. The extent and severity of disease was scored according to the Montreal criteria [23]. A total of sixty-five healthy controls were recruited from the bowel cancer screening programme if they had documented absence of both macroscopic and microscopic inflammation. Subjects were excluded if they received antibiotics within six months prior to recruitment.

Colonic biopsies were obtained from colonoscopy procedures carried out at Aberdeen Royal Infirmary, Aberdeen, UK. Ethical approval for the study was granted by the North of Scotland Research Ethics Service, UK (reference number 04/S0802/8). Written informed consent was obtained from all subjects in the study.

### Biopsy collection, Processing and Genomic DNA Extraction

Biopsy samples were collected from patients with UC and controls during colonoscopy using standard endoscopic forceps (Boston Scientific Nanterre Cedex France). The colonic mucosa was washed with sterile water via the colonoscope to remove residual faecal material. Biopsies were immediately snap-frozen in liquid nitrogen and then transferred to a -80°C freezer for storage pending DNA extraction and analysis.

Genomic DNA was extracted from the biopsies using the QIAamp DNA Mini Kit (Qiagen, Crawley, UK) according to an established modification of the manufacturer's instructions, optimised in-house for colonic biopsy tissue [14]. Biopsy samples were kept frozen until the addition of ATL buffer, thereafter they were allowed to equilibrate to room temperature. An additional 10 µl of Proteinase K was added for an initial lysis period of 18 hours to ensure complete lysis of the biopsy material prior to DNA extraction. DNA obtained from the biopsy samples was initially subjected to universal bacterial PCR to confirm the suitability of the DNA for further analysis [24].

### PCR amplification

#### *Campylobacter* genus specific PCR

The *Campylobacter* genus-specific primers, C412F and C1228 R, described by Linton *et al* in 1996 were used to amplify a ≈800 bp fragment within the 16S rRNA gene of *Campylobacter* species [25]. *Campylobacter* DNA was detected in the extracted biopsy samples by PCR using a 50 µl reaction mixture consisting of 10 pmol of each primer (C412F and C1228R [Sigma-Aldrich, UK]), 1× PCR buffer (Roche, UK), 250 nM of each deoxy-nucleotide-triphosphate (Bioline, UK), 2 mM MgCl2 (Roche, UK), 1 U of Taq polymerase (Roche,UK), and 40 ng of DNA. The PCR cycling conditions used were: 30 cycles of 94°C for 30 seconds, 55°C for 30 seconds, and 72°C for 2 minutes.

#### *Campylobacter concisus* specific PCR

*C. concisus* specific primers, Concisus F and Concisus R, were used to amplify a 560 bp fragment within the 16S rRNA gene of *C. concisus* strains as per the protocol described by Ming Man *et al* in 2010 [18]. The sequences corresponding to the primer pair Concisus F/Concisus R were located within the region amplified by the *Campylobacter* genus specific primer C412F and C1228R and hence a nested PCR approach was used to identify the *C. concisus* strains - *Campylobacter* genus-specific PCR was therefore followed by *C. concisus* specific PCR. The composition of a 50 µl PCR reaction mixture was: 10 pmol of each primer (Concisus F and Concisus R [Sigma-Aldrich, UK]), 1× PCR buffer (Roche, UK), 250 nM of each deoxy-nucleotide-triphosphate (Bioline, UK), 2 mM MgCl2 (Roche, UK) and 1 U of Taq polymerase (Roche, UK). The optimum thermal cycling conditions for the *C. concisus*-specific nested PCR were: 94°C for 5 minutes, 40 cycles of 94°C for 30 seconds, 65°C for 30 seconds, and 72°C for 1 minute, followed by 72°C for 7 minutes.

### Cloning and Sequencing

To enable the identification of other *Campylobacter* species, *Campylobacter* genus positive PCR products were first subjected to restriction fragment length polymorphism (RFLP) analysis by digestion with *Dde* I enzyme [26]. PCR products which did not show a mixed RFLP pattern were directly sequenced on an Applied Biosystems model 3730 automated capillary DNA sequencer using the *Campylobacter* genus specific primers C412F and C1228R. Samples with mixed RFLP profiles were analyzed by cloning the *Campylobacter* genus positive PCR products into JM109 competent cells with pGEM-T-easy vector and the sequence of the insert was established with M13 sequencing primers.

The sequences obtained were compared to those of the National Center for Biotechnology Information GenBank database using the basic local alignment search tool (BLAST) search program (http://www.ncbi.nlm.nih.gov).

Multiple alignments and phylogenetic analyses were performed using Bioedit (version 7.0.5.3) (http://www.mbio.ncsu.edu/BioEdit/bioedit.html) and a dendogram was constructed using MEGA version 4 software [27].

### GenBank Sequence Submission

All 16S rRNA gene sequences derived from either direct sequencing or cloning of *Campylobacter* genus-specific PCR products were submitted to GenBank with the accession numbers from JF795865 to JF795912.

### Statistical Analysis

Statistical analysis was performed using the Pearson Chi Squared, 2-tailed test or the Fisher's exact test wherever appropriate, utilising Graph Pad software (San Diego, CA).

## Results

### Patient characteristics

The prospective UC cohort (n = 69, male 46.4%) had a mean age of 45.3±17.9 years at the time of index colonoscopy. The control group (n = 65, male 59.3%) had a mean age of 61.4±8.5 years at the time of index colonoscopy. There was a statistically significant difference in age between the UC cohort and the control group (p<0.0001). A total of 115 biopsy sites were analysed from UC patients. Twenty four (34.8%) subjects had a single site analysed and 45 (65.2%) had more than one biopsy site assessed. Of the 115 biopsies analysed, 68 (59.1%) were from histologically inflamed sites, whereas 47 (40.9%) were from histologically normal sites A single biopsy site was analysed from each control subject. The clinical characteristics and Montreal classification of the patients with UC are summarised in Table 1.

**Table 1:** Clinical characteristics of subjects with Ulcerative colitis.

	Ulcerative Colitis
**Number of subjects**	69
**Age at diagnosis(Years) ± SD**	45.6±27.8
**Sex (Male %)**	32 (46.4%)
**Montreal classification (Extent)**	
**Proctitis E1 (%)**	8 (11.6%)
**Left sided UC E2 (%)**	44 (63.8%)
**Extensive UC E3 (%)**	17 (24.6%)
**Montreal classification (Severity)**	
**Clinical remission S0 (%)**	7 (10.1%)
**Mild UC S1 (%)**	16 (23.2%)
**Moderate UC S2 (%)**	32 (46.4%)
**Severe UC S3 (%)**	14 (20.3%)

### Detection of *Campylobacter concisus* from mucosal biopsy samples of adults with UC and controls

**Figure 1:**
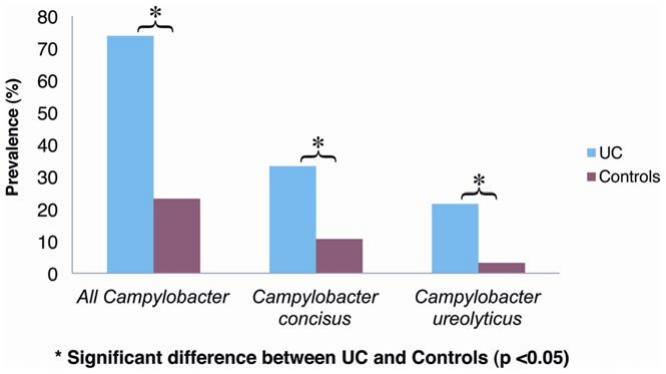
*Campylobacter species*, *Campylobacter concisus* and *Campylobacter ureolyticus* in subjects with ulcerative colitis and controls. The prevalence of all *Campylobacter species* was 73.9% (51/69) in subjects with UC as compared to 23.1% (15/65) in controls (p = 0.0001). *Campylobacter concisus* was detected in 33.3% (23/69) of subjects with UC, which was significantly higher than the prevalence rate of 10.8% (7/65) from controls (p = 0.0019). *Campylobacter ureolyticus* was positive in 21.7% (15/69) of samples from UC subjects as opposed to 3.1% (2/65) in controls (p = 0.0013).

Utilising the species-specific primers, *C. concisus* was detected in 23 of the 69 subjects with ulcerative colitis and 7 of the 65 controls. The prevalence of *C. concisus* in the UC population was 33.3% which was significantly higher than that in the controls, 10.8% (p = 0.0019) (Figure 1).

### Detection of all *Campylobacter* species from mucosal biopsy samples of adults with UC and controls

Utilising *Campylobacter* genus specific primers, *Campylobacter* DNA was detected in 51 of the 69 patients with UC, and 15 of the 65 controls. The prevalence of *Campylobacter* was significantly higher in cases of UC, with a prevalence of 73.9% compared to 23.1% in controls (p = 0.0001). Sequencing of the remaining *Campylobacter* positive samples revealed that *C. ureolyticus* was present in 21.7% (15/69) of samples from UC patients as opposed to 3.08% (2/65) from controls (p = 0.0013) (Figure 1). Detailed breakdown of all the species identified from both UC subjects and controls is summarised in Table 2. Other notable members of the *Campylobacter* genus that were identified include: *Campylobacter hominis*, *Campylobacter curvus*, *Campylobacter gracilis*, *Campylobacter showae* and *Campylobacter jejuni*.

**Table 2:** *Campylobacter* species identified by sequencing.

*Campylobacter* Species Identified	Number of Subjects Combination of Species identified in	
	Ulcerative Colitis (%)	Healthy Control (%)
**Single species identified**		
*Campylobacter concisus*	16 (23.2%)	5 (7.7%)
*Campylobacter ureolyticus*	7 (10.1%)	2 (3.1%)
*Campylobacter hominis*	7 (10.1%)	2 (3.1%)
*Campylobacter curvus*	1 (1.4%)	3 (4.6%)
*Campylobacter gracilis*	1 (1.4%)	0
*Campylobacter showae*	2 (2.9%)	0
*Campylobacter jejuni*	1 (1.4%)	0
**Two or more species co-existing within Subject**		
*Campylobacter ureolyticus* *Campylobacter hominis*	6 (8.7%)	0
*Campylobacter showae* *Campylobacter concisus*	2 (2.9%)	0
*Campylobacter curvus* *Campylobacter concisus*	2 (2.9%)	0
*Campylobacter hominis* *Campylobacter concisus*	1 (1.4%)	2 (3.1%)
*Campylobacter rectus* *Campylobacter concisus*	1 (1.4%)	0
*Campylobacter ureolyticus* *Campylobacter concisus*	1 (1.4%)	0
*Campylobacter hominis* *Campylobacter curvus*	0	1 (1.5%)
*Campylobacter ureolyticus* *Campylobacter jejuni*	1 (1.4%)	0

### Mixed *Campylobacter* species from mucosal biopsy samples of adults with UC and controls

Mixed *Campylobacter* species were more likely in UC patients, 20.3% (14/69) as compared to controls 4.6% (3/65) (p = 0.0084). The most commonly identified combination of *Campylobacter* species in adults with UC was *C.ureolyticus* and *C. hominis* seen in 8.7% of all cases. The most common mix in the controls was *C. concisus* and *C. hominis* accounting for 3.1% of cases. A variety of different *Campylobacter* combinations were noted in UC as opposed to controls wherein only two combinations involving *C. concisus/C. hominis* and *C. hominis/C. curvus* were found (Table 2).

### Prevalence of *Campylobacter* species and *Campylobacter concisus* in relation to gender, site of disease and severity of symptoms

There were no significant differences noted between the prevalence of *Campylobacter* species or indeed *C. concisus* with respect to gender of subjects with UC or controls. The prevalence of all *Campylobacter* species and *C. concisus* with reference to extent and severity of disease is summarised in Table 3. There were no significant differences noted between the prevalence of *Campylobacter* species or specifically *C. concisus* with respect to extent of disease. The detection of *Campylobacter* species and *C. concisus* was also studied in relation to the severity of symptoms according to the Montreal classification, as summarised in Table 3. The only significant difference noted was with the prevalence of all *Campylobacter* species and disease severity between patients with moderate UC (87.5%) and those with severe UC (50%) (p = 0.01). No significant differences were noted between the prevalence of *Campylobacter concisus* with respect to severity of symptoms.

**Table 3:** Distribution of *Campylobacter* species according to extent and severity of disease.

Subjects	Characteristic		Number	*Campylobacter* species	*Campylobacter concisus*
**Ulcerative colitis**	**Montreal extent**	**Proctitis**	8	5 (62.5%)	2 (25%)
		**Left sided UC**	44	35 (79.6%)	15 (34.1%)
		**Pancolitis**	17	11 (64.7%)	6 (35.3%)
	**Montreal severity**	**Remission**	7	4 (57.1%)	2 (28.6%)
		**Mild**	16	12 (75%)	6 (37.5%)
		**Moderate**	32	28 (87.5%)	13 (40.6%)
		**Severe**	14	7 (50%)	2 (14.3%)
**Controls**			65	15(23.1%)	7 (10.8%)

### Prevalence of *Campylobacter* species and *Campylobacter concisus* in relation to inflamed and non-inflamed biopsy samples from subjects with UC

Of the 115 biopsy sites analysed from subjects with UC, 68 were from histologically inflamed sites whereas 47 were from sites that were histologically normal. The prevalence of all *Campylobacter* species was 66.2% (45/68) from inflamed biopsies and 46.8% from histologically normal biopsies (22/47) which was significant (p = 0.038). However, there was no significant difference noted between the prevalence of *Campylobacter concisus* in inflamed biopsies 25% (17/68) and that from normal biopsies 21.3%(10/47) (p = 0.64).

### Comparison of *Campylobacter* and *Helicobacter* species from mucosal biopsies

As previously stated, *Helicobacteraceae* PCR positivity was significantly higher in UC than controls within this cohort: 32 of 69 (46.4%) versus 10 of 65 (15.4%) respectively (p = 0.0002) [14]. The majority of *Helicobacter* species were non*-pylori*, constituting 96.9% (31/32) of all PCR positive subjects with UC and 80% (8/10) of all PCR positive controls. We wanted to compare the previously published *Helicobacter* prevalence with *Campylobacter* positivity from the current study. The prevalence of *Campylobacter* in subjects with UC (51/69) was higher than the prevalence of all H*elicobacter* (32/69) but it did not reach statistical significance (p = 0.252). Similarly, the difference between the prevalence of *Campylobacter* (15/65) and *Helicobacter* (10/65) was not statistically significant in controls (p = 0.374). The prevalence of both these bacterial groups was significantly less in controls as opposed to patients with UC. Identification of mixed *Helicobacter* and *Campylobacter* species was noted in 34.8% of all patients with UC (24/69) and 47.1% (24/51) of the UC patients harbouring *Campylobacter*.

### Phylogenetic Analysis

**Figure 2:**
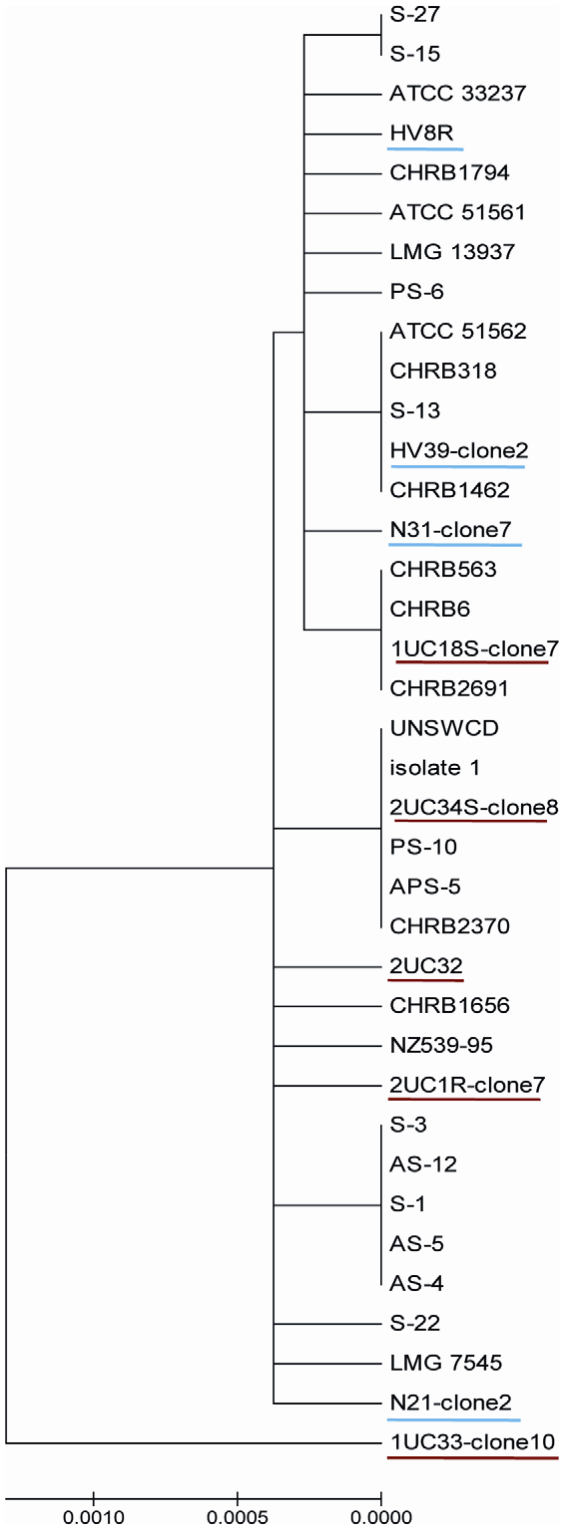
Phylogenetic tree constructed using sequences of the 16S rRNA gene of *C. concisus* strains from UC and controls and other strains available in GenBank. Strains from UC are underlined in red and those from HC are underlined in blue. The evolutionary history was inferred using the Neighbor-Joining method. The bootstrap consensus tree inferred from 500 replicates was taken to represent the evolutionary history of the taxa analysed. The evolutionary distances were computed using the Maximum Composite Likelihood method.

Sequence analysis ≈800 bp *Campylobacter* genus specific PCR amplicons, revealed a high nucleotide sequence similarity to various *Campylobacter* species with a maximum identity of 99–100%. *C. concisus* sequences analysed from the UC and HC group did not cluster into separate groups in the dendogram as shown in Figure 2.

## Discussion

This study has investigated the prevalence of the *Campylobacter* genus of bacteria in UC and for the first time, a positive association has been noted between the presence of *Campylobacter* species and adult UC. *Campylobacter* was detected in a significantly larger proportion of UC patients as opposed to controls. Furthermore, two individual species, namely *C. concisus* and *C. ureolyticus* were found to have a significant association with UC. The control group comprised of a significantly older group of patients as they had been recruited from the bowel screening programme. A recent study found that the greatest incidence of *Campylobacter* infection was for those aged more than 60 years of age, making this difference even more clinically significant [28]. A study documenting prevalence of *C. concisus* in salivary samples from different age groups did not find any significant difference among various adult age groups but found the prevalence to be significantly lower in the children age 3–5 years [29].

In the last two years, *C. concisus* has been associated with paediatric CD, with two studies documenting this association from faecal and biopsy samples [17], [18]. The reported prevalence of *C. concisus* from these two studies was 65% from faecal samples and 51% from biopsy samples. The prevalence of *C. concisus* in biopsy samples from adult UC in our study was lower than these studies at 31.9%. Our finding contrasts with these previous studies as it is based on adult subjects with UC as opposed to children with CD. Therefore, our findings have expanded the role of *C. concisus* to encompass both forms of IBD necessitating further studies to firstly validate our findings and secondly to delineate the exact mechanism of this association.

This study not only found an increased prevalence of *C. concisus* in mucosal biopsy samples from adults with UC but also identified DNA from this putative pathogen in one in ten controls. *C. concisus* is a common commensal in the oral mucosa and it has been associated with periodontitis [30]. A study by Zhang *et al* aimed to look at the relative rates of isolation and detection of *C. concisus* from salivary samples of healthy controls and patients with IBD but did not find any significant difference between the two groups (97% vs. 100%) [29]. Protein profiling of six of the oral strains were compared with an intestinal strain of *C. concisus* using SDS-PAGE. Interestingly only one of the six oral strains matched the intestinal strain, suggesting that different strains of the bacteria may exist in the oral cavity with some having the ability to colonise the intestine. The increased detection of this oral commensal in UC may be as a result of defective innate immunity, allowing easier access to an additional ecological niche where the organism may potentially cause disease. This hypothesis is supported by the detection of this pathogen during episodes of diarrhoea in immunocompromised adults [20]. Additionally, the finding of this bacterium in the extremes of age groups, who are characterized with poorer immunity, suggests that *C. concisus* may be an opportunistic pathogen [31].

There is an additional suggestion that *C. concisus* exists as a heterogeneous population with both pathogenic and non-pathogenic strains co-existing together, with disease being manifest following infection with a pathogenic genotype. This has been elegantly demonstrated by Aabenhus *et al* who utilized amplified fragment length polymorphism (AFLP) analysis from a variety of clinical isolates of *C. concisus* obtained from sixty-two immunocompetent and immunocompromised individuals over a period of five years [32]. Their analysis showed at least four distinct *C. concisus* genomospecies which exhibited differences in their spectra of virulence potential. In another similar study, analysis of SDS-PAGE protein profiles and PCR amplification of 23S rDNA assigned clinical *C. concisus* isolates into two distinct, but discordant groups [33]. Identification of two genetically distinct clusters have also been reported from a recent report utilizing analysis of amplified fragment length polymorphism profiles [34]. This study identified genomospecies A from healthy individuals and genomospecies B from patients with diarrhoea. More importantly they found that the pathogenic strains (genomospecies B) displayed greater epithelial invasion and translocation. This is possibly the first report of a genotype-phenotype correlation of pathogenicity of *C. concisus* isolates. During the current study, phylogenetic analysis was also undertaken to see if a similar clustering effect could be seen based on 16S rDNA sequence data. Our study cohort did not demonstrate a similar clustering effect, indicating that 16S rDNA sequence data is not as discriminatory as 23S rDNA for categorising *C. concisus* isolates. The debate on whether differing phenotypic characteristics of *C. concisus* can be identified by studying genetic composition still needs to be resolved.

In comparison to *C. concisus*, *C. ureolyticus* is a relatively unknown gastrointestinal pathogen. In its previous nomenclature as *Bacteroides ureolyticus* it was known to cause soft tissue infections and urethritis and it has only recently been implicated as a cause of diarrhoea [22], [35], [36]. Its significant association with UC in our study suggests that these patients may be susceptible to colonisation with *Campylobacter* and perhaps this is genus-specific rather than to any particular *Campylobacter* species. This is supported by the increased number of mixed *Campylobacter* (with relatively rare members of the *Campylobacter* genus, including *C. showae*, *C. curvus* and *C. gracilis*) in adults with ulcerative colitis as opposed to controls. This finding is similar to those in paediatric Crohn's disease reported by Man et al [18]. The increased diversity of *Campylobacter* species in UC may reflect a specific defect in the immunological handling of this genus in the intestinal mucosa of UC. The additional finding of mixed *Helicobacteraceae* and *Campylobacter* species in UC suggest that this defect may extend to the entire phylum of *Proteobacteria*.

A sub-group analysis of the patients with UC was performed which found no definite relationship between extent of disease and the prevalence of *Campylobacter* and specifically *C. concisus*. There was a significantly lower prevalence of all *Campylobacters* in subjects with severe colitis (50%) as opposed to moderate colitis (87.5%), but no such difference was noted with the prevalence of *C. concisus* infection. The obvious corollary to this finding is that our study did not have the power to detect differences between the various sub-groups of UC. This finding appears to be against the obvious premise that a greater exposure should generally lead to greater incidence of the effect. Contrary to this finding, a greater prevalence of *Campylobacter* species was noted in histologically inflamed biopsies as opposed to normal biopsy samples taken from UC patients. One can surmise that there can be regional changes in mucosal bacterial species, in this case *Campylobacter*, that may trigger an inflammatory cascade which then leads to ulceration and loss of the epithelial surface that harbours these organisms. This would paradoxically lead to reduced identification of the pathogen in the most severe phases of active disease. Future studies should also be conducted on non-IBD inflammatory colonic diseases to ensure that the presence of the bacteria is not merely a superinfection of inflamed tissue.

In the classic essay by Bradford Hill on the theory of causation he states that: “We must not be too ready to dismiss a cause and effect hypothesis merely on the grounds that the observed association appears to be slight" [37]. This doctrine is all the more relevant when considering the role of a solitary pathogen like *C. concisus* in the broader aetiopathogenesis of a multifactorial disease like UC. The pathogenic potential of *C. concisus* has been elegantly demonstrated in an *in-vitro* model wherein these strains were demonstrated to be invasive and also induced production of pro-inflammatory cytokines from epithelial cells, monocytes and macrophages [38]. A cytolethal distending toxin (CDT)-like effect on Vero cells has been shown by clinical isolates of *C. concisus* from subjects with diarrhoea [33]. The bacterium also produces cell-associated and secreted haemolysins that may have a role in pathogenicity [39]. It is obvious that this bacterium can cause tissue damage but whether it is the initiating trigger, the perpetuating factor or merely an epiphenomenon amidst the mucosal inflammatory cascade is a question that still needs to be answered.
